# Computational Drug Repurposing for Antituberculosis Therapy: Discovery of Multi-Strain Inhibitors

**DOI:** 10.3390/antibiotics10081005

**Published:** 2021-08-19

**Authors:** Valeria V. Kleandrova, Marcus T. Scotti, Alejandro Speck-Planche

**Affiliations:** 1 Laboratory of Fundamental and Applied Research of Quality and Technology of Food Production, Moscow State University of Food Production, Volokolamskoe shosse 11, 125080 Moscow, Russia; valeria.kleandrova@gmail.com; 2 Postgraduate Program in Natural and Synthetic Bioactive Products, Federal University of Paraíba, João Pessoa 58051-900, Brazil; mtscotti@gmail.com

**Keywords:** artificial neural networks, drug repurposing, ensemble, MLP, QSAR, tuberculosis, strain, virtual screening

## Abstract

Tuberculosis remains the most afflicting infectious disease known by humankind, with one quarter of the population estimated to have it in the latent state. Discovering antituberculosis drugs is a challenging, complex, expensive, and time-consuming task. To overcome the substantial costs and accelerate drug discovery and development, drug repurposing has emerged as an attractive alternative to find new applications for “old” drugs and where computational approaches play an essential role by filtering the chemical space. This work reports the first multi-condition model based on quantitative structure–activity relationships and an ensemble of neural networks (mtc-QSAR-EL) for the virtual screening of potential antituberculosis agents able to act as multi-strain inhibitors. The mtc-QSAR-EL model exhibited an accuracy higher than 85%. A physicochemical and fragment-based structural interpretation of this model was provided, and a large dataset of agency-regulated chemicals was virtually screened, with the mtc-QSAR-EL model identifying already proven antituberculosis drugs while proposing chemicals with great potential to be experimentally repurposed as antituberculosis (multi-strain inhibitors) agents. Some of the most promising molecules identified by the mtc-QSAR-EL model as antituberculosis agents were also confirmed by another computational approach, supporting the capabilities of the mtc-QSAR-EL model as an efficient tool for computational drug repurposing.

## 1. Introduction

Tuberculosis (TB) constitutes the deadliest infectious disease that afflicts humankind. The causative agent of TB, *Mycobacterium tuberculosis* (*Mtb*), was responsible in 2018 for causing more than 10 million cases of active TB, resulting in 1.5 million deaths [1]. Despite the efforts of the scientific community in providing anti-TB therapies through the process known as drug discovery, several aspects pose great challenges for the safe and successful eradication of TB. On one hand, the thick cell wall formed by mycolic acids, the ability of certain enzymes to modify/inactivate drug molecules, the presence of drug efflux systems, and the occurrence of spontaneous mutations in the bacterial genome are the main biological attributes that make *Mtb* considerably resistant (multidrug-resistant TB) to current anti-TB treatments [2]. On the other hand, the FDA-approved anti-TB drugs are associated with a low diversity of mechanisms of action [3] and exhibit a wide range of side effects [4]. All these ideas, together with the complexity and remarkable expenditure of time and financial resources during the drug discovery process [5], demonstrate that TB is hard to treat and that efficacious anti-TB agents are urgently needed.

A plausible way to tackle the TB problem is to apply the drug repurposing philosophy, which focuses on finding new applications for “old” drugs [6]. In this sense, in the context of identifying novel anti-TB agents, computational approaches have played an essential role by filtering the chemical space, providing different tools that can speed up the discovery of molecules able to inhibit the growth of *Mtb* [7,8,9,10,11,12,13,14]. However, all the computational approaches reported to date have at least one of the following aspects that prevent their full exploitation in virtual screening scenarios: (a) the anti-TB activity is predicted in a generic manner or by considering either only one target protein or *Mtb* strain, (b) small datasets of structurally related chemicals are used to build the computational models, (c) there is no information regarding the experimental protocol or the assay time employed to assess the inhibitory activity against *Mtb*, and (d) no insights are provided concerning the physicochemical properties and structural features that are required in a molecule to have anti-TB activity.

To solve the aforementioned limitations, several researchers have emphasized the use of interpretable in silico models focused on a combination of perturbation theory concepts and machine learning techniques (PTML) [15,16,17], which can integrate different sources of chemical and biological data, enabling the simultaneous prediction of multiple biological endpoints against many targets of varying degrees of complexity. Seminal works on PTML models have found successful applications in diverse research areas such as infectious diseases [18,19], oncology [20,21], neuroscience [22,23,24,25], proteomics [26], metabolomics [27], nanotechnology [28,29,30,31], toxicology [32], and immunology and immunotoxicity [33,34].

Bearing in mind all the previous ideas, we report in this work a special case of the PTML modeling methodology in the context of the search for novel anti-TB chemical therapies, establishing the theoretical foundations for the computational repurposing of drugs as anti-TB agents. Thus, we have developed here a multi-condition model based on quantitative structure–activity relationships and an ensemble of neural networks (mtc-QSAR-EL) able to perform virtual screening for the identification of multi-strain inhibitors of *Mtb*. We also provide the physicochemical and fragment-based structural interpretation of the different molecular descriptors used to build the mtc-QSAR-EL model. Finally, we performed a virtual screening of a large and heterogeneous dataset of agency-regulated chemicals, identifying both already proven anti-TB agents and promising chemical structures with the potential to inhibit *Mtb*.

## 2. Results and Discussion

### 2.1. Performance of the Mtc-QSAR-EL Model and Applicability Domain

The best mtc-QSAR-EL model found by us has twelve *D*[*LQI*]*cj* descriptors, six of them based on hydrophobic factors, five containing steric information, and one focused on polar features of the molecules. Additionally, in terms of atom types, five *D*[*LQI*]*cj* descriptors were based on the effect of halogen atoms, three indicating the influence of heteroatoms (mainly N, O, S, and P), two characterizing the presence of methyl groups, and two accounting for the importance of aromatic carbons. The details regarding each *D*[*LQI*]*cj* descriptor appear in Table 1, while all the molecular descriptors of the chemicals and the corresponding biological data are reported in Supplementary Material 1.

The most appropriate mtc-QSAR-EL model developed here was an ensemble formed by three MLP networks whose parameters are represented in Table 2. These MLP networks have different numbers of neurons in the hidden layer and require diverse error functions and different numbers of epochs to be trained. Two of these MLP networks (first and third) have the same type of activation function (hyperbolic tangent), with the other one having a logistic function. In the output layer, the first and second MLP networks use a softmax function, while the third one uses a logistic function. The combination of these aspects resulted in a difference in performance among these MLP networks, particularly in the case of the sensitivities [*Sn*(%)]*ta*, [*Sn*(%)]*bs*, and [*Sn*(%)]*ap*, as well as the specificities [*Sp*(%)]*ta*, [*Sp*(%)]*bs*, and [*Sp*(%)]*ap*. These six local metrics were of paramount importance in assessing the classification performance of the mtc-QSAR-EL model in both trained and unseen data (training and test sets, respectively) when considering the dissimilar experimental conditions *cj* (see Section 3 for full explanation) under which the molecules were assayed.

The mtc-QSAR-EL model exhibited accuracies [*Ac*(%)] of 93.41% and 85.79% in the training and test sets, respectively. Furthermore, the global statistical indices depicted in Table 3 support the good general performance of the mtc-QSAR-EL model. For instance, the sensitivity *Sn*(%) and the specificity *Sp*(%) have values higher than 90% in the training set. These two global statistical indices reached values higher than 85% in the test set. Additionally, the *MCC* values are greater than 0.7, and, given their closeness to the ideal value of one, we can infer that there is very strong convergence between the observed [*ATB_i_*(*cj*)] and predicted [*Pred*_*ATB_i_*(*cj*)]] values of the categorical variable of anti-TB activity against the different *Mtb* strains. The classification results for all the molecules of our dataset are reported in Supplementary Material 2. The file of the MLP networks can be obtained upon request to the authors.

At the local level, the mtc-QSAR-EL model also had a good performance. In the case of the training set, the local metrics [*Sn*(%)]*ta*, [*Sn*(%)]*bs*, [*Sn*(%)]*ap*, [*Sp*(%)]*ta*, [*Sp*(%)]*bs*, and [*Sp*(%)]*ap* were in the range 70–100%. The only exception was the assay time, 4 d (four days), which exhibited [*Sp*(%)]*ap* = 56.25%. In the case of the test set, a similar result was achieved since the same six statistical metrics were in the interval 71–100%, except for [*Sp*(%)]*ap* = 50% (assay time of four days) and [*Sn*(%)]*ta* = 64% (assay time of five days), as well as [*Sp*(%)]*bs* and [*Sp*(%)]*ap*, with values of 61.54% for the strain *Mtb* (H37Rv_NRF) and the assay protocol “LORA method”, respectively. The previously mentioned global statistical indices and the local metrics discussed here confirm the internal quality and predictive power of the mtc-QSAR-EL model. All the values of [*Sn*(%)]*ta*, [*Sn*(%)]*bs*, [*Sn*(%)]*ap*, [*Sp*(%)]*ta*, [*Sp*(%)]*bs*, and [*Sp*(%)]*ap* can be found in Supplementary Material 2.

**Figure 1:**
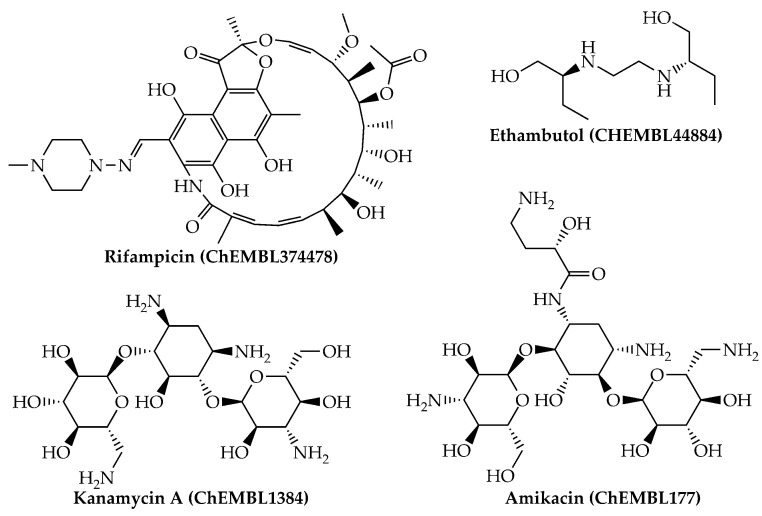
Anti-TB drugs (polyketides, ethylenediamine derivatives, and aminoglycosides) present in our dataset which were correctly classified by the mtc-QSAR-EL model.

**Figure 2:**
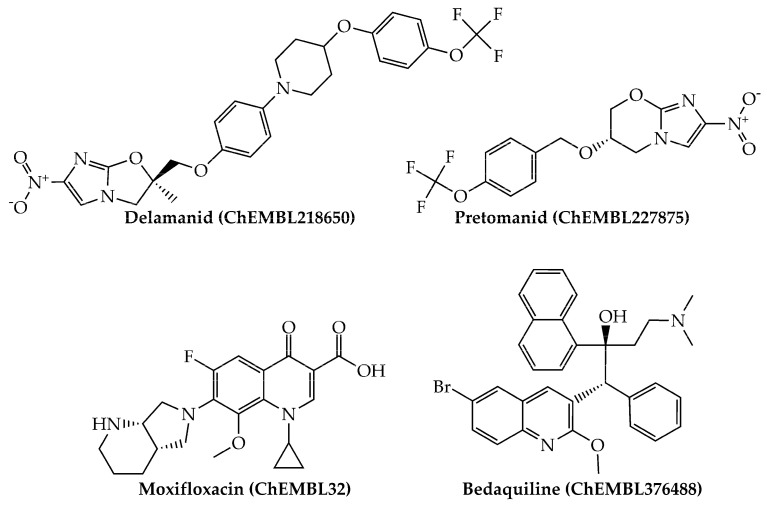
Other anti-TB drugs (nitroimidazopyrans, fluoroquinolones, and diarylquinolines) present in our dataset which were also correctly classified by the mtc-QSAR-EL model.

Last, we would like to highlight that, from a physicochemical and structural point of view, the present mtc-QSAR-EL model classified anti-TB drugs belonging to a wide spectrum of chemical families (Figure 1 and Figure 2) such as polyketides, ethylenediamine derivative, aminoglycosides, nitroimidazopyrans, fluoroquinolones, and diarylquinolines.

Such heterogenicity of chemical structures together with the different definitions of the *D*[*LQI*]*cj* descriptors, the size of the dataset, and the relatively high values of the global and local statistical indices points to the capability and appropriateness of the mtc-QSAR-EL model to predict the anti-TB activity of structurally dissimilar chemicals against the different *Mtb* strains.

Regarding the AD of the mtc-QSAR-EL model, as reported in seminal references (see Section 3.3), we calculated the so-called total score of applicability domain (TSAD), which is derived from the descriptors’ space approach. Since our mtc-QSAR-EL model contained twelve *D*[*LQI*]*cj* descriptors, only those chemicals with TSAD = 12 were considered to be inside the AD (Supplementary Material 3). By carefully inspecting our dataset, we found that only 15 out of 1571 molecules/cases in the dataset were outside the AD of the mtc-QSAR-EL model, 14 of them with TSAD = 11 and one with TSAD = 10. However, 13 out of these 15 seemingly atypical chemicals were correctly classified by considering the consensus prediction approach performed by the mtc-QSAR-EL model. We decided to keep these chemicals in the dataset since the consensus predictions constituted the priority over the descriptors’ space approach to define the AD.

### 2.2. Molecular Descriptors and Their Physicochemical and Structural Meanings

**Figure 3:**
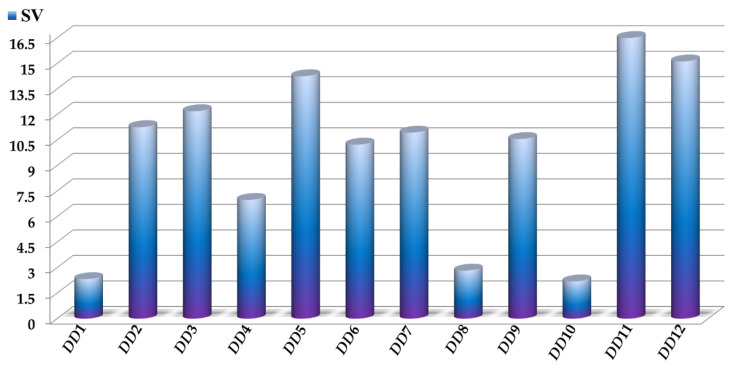
Molecular descriptors present in the mtc-QSAR-EL model and their relative influences assessed as sensitivity values (*SV*). For the sake of simplicity, we used the following abbreviations: *DD*1 = *D*[*LASSq*3(*HYD*)*G*]*ta*, DD2 = *D*[*LASSq*0(*POL*)*G*]*ta*, DD3 = *D*[*LASSq*0(*HYD*)*Y*]*ta*, DD4 = *D*[*LASSq*1(*PSA*)*Y*]*ta*, DD5 = *D*[*LASSq*0(*HYD*)*M*]*bs*, DD6 = *D*[*LASSq*0(*AW*)*P*]*bs*, DD7 = *D*[*LASSq*2(*HYD*)*Y*]*bs*, DD8 = *D*[*LASSq*2(*HYD*)*G*]*ap*, DD9 = *D*[*LASSq*5(*AW*)*G*]*ap*, DD10 = *D*[*LASSq*3(*KU*)*G*]*ap*, DD11 = *D*[*LASSq*2(*HYD*)*M*]*ap*, and DD12 = *D*[*LASSq*2(*AW*)*P*]*ap*.

The sensitivity values *SV* of the *D*[*LQI*]*cj* descriptors are depicted in Figure 3, where the highest values indicate those *D*[*LQI*]*cj* descriptors with the greatest influence and discriminatory power in the mtc-QSAR-EL model. Simultaneously, the comparison between the class-based means for each *D*[*LQI*]*cj* descriptor represented in Table 4 shows us the type of variation that the value of a given *D*[*LQI*]*cj* descriptor should undergo to potentiate the anti-TB activity against the different *Mtb* strains.

As mentioned before, in our mtc-QSAR-EL model, we have six *D*[*LQI*]*cj* descriptors characterizing information regarding atomic hydrophobic contributions. In this sense, we would like to highlight that such contributions are based on the hydrophobicity scale proposed by Ghose and co-workers [35]. According to this scale, aliphatic carbon atoms present negative hydrophobicity values, excluding the fragments of the form *CHX*3, *CR*2*X*2, *CRX*3, and *CX*4 (*X* = O, N, S, P, Se, or halogen). Nitrogenated and oxygenated functional groups also have negative hydrophobic contributions except for the pyrrolic nitrogen (oxygen in furan) atom, *Ar*–NH–*Ar* (and its oxygenated counterpart), with *Ar* being an aromatic (or heteroaromatic ring), and all the tertiary amines.

With that being said, the results presented in Table 4 indicate that the anti-TB activity through the inhibition of different *Mtb* strains can be enhanced by increasing the value of *D*[*LASSq*3(*HYD*)*G*]*ta*, which describes the augmentation of the product of the hydrophobic contributions of any two atoms (with at least one of them being a halogen) that are placed at the topological distance (number of bonds between two atoms without considering bond multiplicity) of three. Examples of fragments with this characteristic are the 5-(halomethyl)pyrimidines, including those with substitutions in positions 4 and/or 6. Notice that *D*[*LASSq*3(*HYD*)*G*]*ta* is the eleventh most important *D*[*LQI*]*cj* descriptor in the mtc-QSAR-EL model. We also have *D*[*LASSq*0(*HYD*)*Y*]*ta* whose increase is directly proportional to the number of heteroatoms in a molecule. In this sense, the presence of fragments such as *Ar*–NO_2_ (*Ar* = aromatic or heteroaromatic ring), primary amines, amides, hydroxyl groups (alcohol), thiols, thioethers, functional groups containing sulfur (with sp2 hybridization) attached to a carbon atom, phosphite, and R3–P = X (R = any group link though carbon, X = O or S) will considerably increase the value of *D*[*LASSq*0(*HYD*)*Y*]*ta* (the fourth most important *D*[*LQI*]*cj* descriptor), favoring the anti-TB activity. The inhibitory activity against the *Mtb* strains seems to be enhanced by the augmentation of the number of methyl groups in the molecules, and such a structural variation is characterized by *D*[*LASSq*0(*HYD*)*M*]*bs*, which is the third most significant *D*[*LQI*]*cj* descriptor in the mtc-QSAR-EL model. We also have *D*[*LASSq*2(*HYD*)*Y*]*bs* (ranked the sixth most influential *D*[*LQI*]*cj* descriptor), which indicates the increase in the product of the hydrophobic contributions of any two atoms (with at least one of them being a heteroatom) that are placed at the topological distance of two. Substructures such as pyrimidin−2-amine, 2-alkylpyrimidines, and urea, as well as aliphatic chains (or alicyclic moieties) attached to hydroxyl, amino, amide, and sulfonamide groups, will favorably increase the value of *D*[*LASSq*2(*HYD*)*Y*]*bs*, with the subsequent improvement in the anti-TB activity. The presence of halogens seems to be of great importance in the increase in the inhibitory activity against the *Mtb* strains, and as in the case of *D*[*LASSq*3(*HYD*)*G*]*ta* (commented above), *D*[*LASSq*2(*HYD*)*G*]*ap* also positively contributes through the increment in the product of the hydrophobic contributions of any two atoms (with at least one of them being a halogen) that are placed at the topological distance of two. Thus, 5-halopyrimidines and 4,6-disubstituted halobenzene fragments increase the value of *D*[*LASSq*2(*HYD*)*G*]*ap*, which is the tenth most significant *D*[*LQI*]*cj* descriptor. Last, we have *D*[*LASSq*2(*HYD*)*M*]*ap* as the most important *D*[*LQI*]*cj* descriptor in the mtc-QSAR-EL model. In this sense, *D*[*LASSq*2(*HYD*)*M*]*ap* involves the increase in the hydrophobic contributions of any two atoms (with at least one of them being a carbon from a methyl group) that are placed at the topological distance of two. Moieties such as 2-methylpyrimidines, as well as aliphatic chains (or cycloalkane rings) containing methyl groups, amides, methoxy groups, and toluene fragments, will increase the value of *D*[*LASSq*2(*HYD*)*M*]*ap*, and therefore the anti-TB activity against the diverse *Mtb* strains reported here.

Our mtc-QSAR-EL model also has five *D*[*LQI*]*cj* descriptors associated with steric factors. In this context, *D*[*LASSq*0(*POL*)*G*]*ta* describes the diminution of the polarizability of the halogens. The value of *D*[*LASSq*0(*POL*)*G*]*ta* (having the fifth highest influence) can be decreased either by diminishing the number of halogens or by the presence of fluorine atoms. Consequently, halogens are usually important for the activity profiles of the molecules, and the case of the anti-TB activity is not an exception. Thus, functional groups such as trifluoromethyl (and, to a lesser degree, dichloromethyl and bromomethyl), 1,2-difluorobenze, and 2-chlorobenze will decrease the *D*[*LASSq*0(*POL*)*G*]*ta* value enough to favor the inhibitory potency against any *Mtb* strain. Two of the five steric *D*[*LQI*]*cj* descriptors characterize the presence of the aromatic carbons in the molecules. On one hand, the increase in the value of *D*[*LASSq*0(*AW*)*P*]*bs* is directly proportional to the increase in the number of aromatic carbons (e.g., benzene rings and polycyclic substructures such as naphthalene), while *D*[*LASSq*2(*AW*)*P*]*ap*, in addition to benefiting from the presence of aromatic carbons, is also favored by the presence of relatively heavy atoms (Cl, Br, and S) placed at the topological distance of two with respect to an aromatic carbon. Therefore, fragments that also increase the value *D*[*LASSq*2(*AW*)*P*]*ap* (and, to a lesser degree, *D*[*LASSq*0(*AW*)*P*]*bs*) are halobenzene and benzenethiol. The descriptors *D*[*LASSq*0(*AW*)*P*]*bs* and *D*[*LASSq*2(*AW*)*P*]*ap* rank eighth and second among the most important *D*[*LQI*]*cj* descriptors, respectively. Two other steric *D*[*LQI*]*cj* descriptors take into account the presence of halogens. One of them is *D*[*LASSq*5(*AW*)*G*]*ap* (exhibiting the seventh highest influence), which characterizes the increase in the atomic weight of any two atoms (with at least one of them being a halogen) placed at the topological distance of five or less. Fragments of the type ZCX3 (Z = any atom, X = Cl or Br) and 1,4-dihalobenze can favorably increase the value of *D*[*LASSq*5(*AW*)*G*]*ap*. The other steric *D*[*LQI*]*cj* descriptor is *D*[*LASSq*3(*KU*)*G*]*ap* and expresses the increment in the atomic accessibility (ability to interact with atoms from other molecules) of any two atoms (with at least one of them being a halogen) placed at the topological distance of three. In this case, 1,2-dihalobenzene substructures (Cl and Br favored over F) and moieties such as ZCX3, 5-halopyrimidines, and 4,6-disubstituted halobenzene are examples of fragments that increase the value of *D*[*LASSq*3(*KU*)*G*]*ap*. In the mtc-QSAR-EL model, *D*[*LASSq*3(*KU*)*G*]*ap* is ranked twelfth among all the *D*[*LQI*]*cj* descriptors.

Finally, we have *D*[*LASSq*1(*PSA*)*Y*]*ta* (with the ninth highest significance) characterizing the increase in the polar surface area of any two adjacent heteroatoms, and, therefore, only pyridazine and 1,2,3-triazine rings, as well as the sulfonamide and phosphorus-containing functional groups (phosphorus forming bonds with only oxygen and/or sulfur), will considerably augment the value of *D*[*LASSq*1(*PSA*)*Y*]*ta*.

### 2.3. Computational Drug Repurposing of Agency-Regulated Chemicals as Anti-TB Agents

We performed the virtual screening of a dataset formed by 8898 agency-regulated chemicals (Supplementary Material 4), which included (but was not limited to) investigational and FDA-approved drugs. These were predicted by considering the 24 experimental conditions *cj* reported in the dataset used to build the mtc-QSAR-EL model, yielding 213,552 predictions (Supplementary Materials 5 and 6). In terms of the reliability of the predictions using the AD of the mtc-QSAR-EL model according to the descriptors’ space approach, we generated the TSAD values for each of these 8898 chemicals (Supplementary Material 7). Then, the metrics FA(%) and S(TSAD) were calculated (see Section 3.5 for full explanation). In any case, we would like to highlight that FA(%) describes the anti-TB potential of a molecule because it expresses the frequency in which a chemical is predicted as active against *Mtb*. A high FA(%) value (maximum value is 100%) for a chemical means that it has a higher probability of having anti-TB activity by inhibiting multiple *Mtb* strains with MIC_90_ < 7622.22 nM, which is the highest of the MIC_90_ cutoffs reported in this work (see Section 3.1). At the same time, a high S(TSAD) value (the ideal value = 12 × 24 experimental conditions *cj* = 288, with 12 being the maximum TSAD value) indicates the general tendency of a chemical to be inside the AD according to the descriptors’ space approach, which, together with the consensus predictions performed by the mtc-QSAR-EL model, helps to assess the degree of reliability of such predictions.

The combined use of FA(%) and S(TSAD) (Supplementary Material 8) allowed us to rank the 8898 agency-regulated chemicals, and those with FA(%) > 80% and S(TSAD) ≥ 276 were the top ranked, giving priority to those exhibiting the highest FA(%) values. Notice that there is no “rule of thumb” in terms of the criteria used to prioritize chemicals. Therefore, in the present study, we employed these rigorous cutoff values for FA(%) and S(TSAD) to achieve a virtual screening hit rate of 0.49% (44 out of 8898 chemicals) which is higher than the greatest hit rate value of 0.14% for high-throughput screening but lower than smallest hit rate value for virtual screening campaigns (1%) [36].

**Figure 4:**
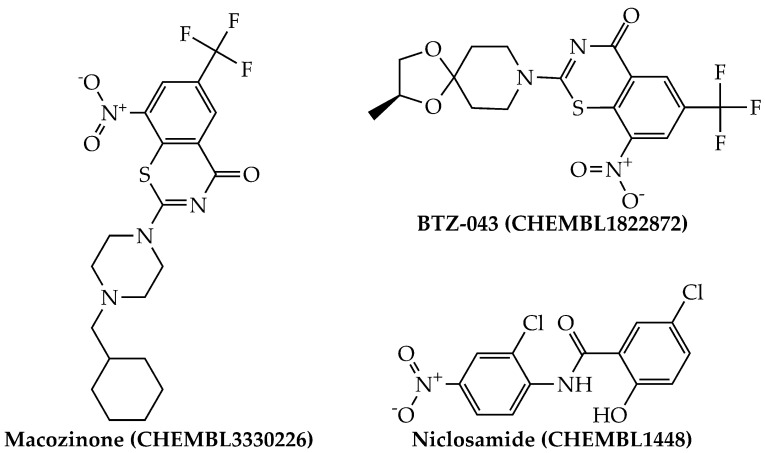
Structures of the agency-regulated chemicals with a proven anti-TB activity identified by the mtc-QSAR-EL model.

By using the aforementioned metrics for compound prioritization, the mtc-QSAR-EL model identified three chemicals with experimentally proven anti-TB activity (Figure 4): macozinone (PBTZ-169), BTZ-043, and niclosamide. Notice that macozinone is a remarkably potent piperazine-containing benzothiazinone, being able to inhibit multiple *Mtb* strains at MIC_99_ ≤ 1 nM [37]. In the case of BTZ-043, although structurally related to macozinone, it lacks the 2-(4-(cyclohexylmethyl)piperazin-1-yl) moiety, which decreases its activity. Still, BTZ-043 is a nanomolar inhibitor of several *Mtb* strains [37,38]. On the other hand, niclosamide offers very attractive opportunities for anti-TB therapies because, in addition to being a recognized antihelminthic drug, it also has a wide-spectrum antimicrobial profile, which includes nanomolar to micromolar potency against diverse viruses (including coronaviruses) [39], as well as anti-TB activity in the low micromolar range [40,41].

We should recall that the cutoff values of the metrics FA(%) and S(TSAD) used by us are very rigorous. If, for instance, we slightly relax these two metrics (e.g., FA(%) > 60% and S(TSAD) ≥ 270), other agency-regulated chemicals with experimentally proven anti-TB activity pop up. These are the cases of biapenem (FA(%) = 66.67% and S(TSAD) = 288) and TBA-354 (FA(%) = 91.67% and S(TSAD) = 270), whose anti-TB profile has been demonstrated in vitro (low micromolar range) and in vivo [42,43]. Notice that further relaxing FA(%) and/or S(TSAD) will allow the mtc-QSAR-EL model to identify more anti-TB agents, but a larger number of false positives may also be predicted. In the end, it is up to the analyst to select the adequate values of the metrics FA(%) and S(TSAD), which will depend on the number of drugs available for testing, with this being a key element when planning the expenditure of financial resources for experimental validation of virtually selected chemicals. In any case, we advise the use of FA(%) > 29% and S(TSAD) ≥ 250 since with these cutoffs, most of the known FDA-approved and investigational anti-TB drugs (not included in the dataset used to build our mtc-QSAR-EL model) were identified in the virtual screening analysis. We would like to highlight that the FA(%) value suggested by us is in the range reported for the hit rate in the prospective virtual screening [36,44].

Returning to the top 44 molecules predicted by the mtc-QSAR-EL model from the 8898 agency-regulated chemicals, we ran an experiment. To obtain a deeper insight regarding the new molecular patterns with promising anti-TB potential, we used the webserver mycoCSM [45], which is an online tool with the capability to predict the antimycobacterial profile of any molecule, including the anti-TB activity. The results of the top 44 agency-regulated chemicals identified as potential anti-TB agents (multi-strain inhibitors of *Mtb*) by our mtc-QSAR-EL model together with the predictions derived from mycoCSM appear in Table 5. It can be seen that the mtc-QSAR-EL model and the webserver mycoCSM converge in 10 out of 44 chemicals (22.73%). In our opinion and experience, such a convergence is very good taking into account that the mtc-QSAR-EL model and the webserver mycoCSM employed dissimilar approaches to characterize the molecular structure, different machine learning algorithms, and distinct ways to consider experimental information when building the computational models. In the end, given all the experimental and theoretical evidence, we can conclude that our mtc-QSAR-EL model can be efficiently used alone or in combination with other in silico tools for virtual screening of anti-TB molecules, which may inhibit several *Mtb* strains.

## 3. Materials and Methods

### 3.1. Dataset and Computation of the Molecular Descriptors

All the chemical and biological data associated with the anti-TB activity were retrieved from the public database known as ChEMBL [46,47,48]. The dataset used in the present study was formed by 1237 molecules belonging to different chemical families. These molecules were experimentally tested for their inhibitory activity against *Mtb* and where the MIC_90_ (minimum inhibitory concentration that prevents the visible growth in 90% of the *Mtb* isolates) was measured. More specifically, each molecule was assayed by considering at least 1 out of 7 assay times (*ta*), against at least 1 out of 8 *Mtb* strains (*bs*), and involving at least 1 out of 4 assay protocols (*ap*). Notice that each combination of the elements *ta*, *bs*, and *ap* represents a unique experimental condition *cj*, which can be expressed as *cj*(ta, *bs*, *ap*). If a molecule was found to be assayed more than one time by considering the same experimental condition *cj*, the duplicate data were deleted, and we kept the lowest MIC_90_ value for that molecule. If two stereoisomers were tested under the same experimental condition *cj*, we kept only the stereoisomer with the lowest MIC_90_ value. In any case, in our dataset, most of the molecules were tested by considering only one *cj*, and, therefore, after removing entries with lacking SMILES codes, values, units, duplicates, and unclear information regarding the assay time or the test protocol, the dataset ended up having 1571 cases. Each case/molecule in the dataset was annotated as active (*ATB_i_*(*c_j_*) = 1) or inactive (*ATB_i_*(*c_j_*) = −1), where *ATB_i_*(*c_j_*) was a dichotomous variable indicating the anti-TB activity of the *ith* case/molecule under the experimental condition *c_j_*. Assignments of active and inactive cases were realized by considering the different MIC_90_ cutoff values depending on the assay time (Table 6).

Several reasons justified the selection of the MIC_90_ cutoffs in Table 6. First, by inspecting our dataset formed by the 1571 cases, one can see that there are FDA-approved anti-TB drugs (isoniazid, rifampicin, ethambutol, etc.) with MIC_90_ values that fall either above or below the cutoffs, which means that if a query chemical is predicted as active (or inactive) by the mtc-QSAR-EL model, it will be possible to assess the differences between the inhibitory potential of that query molecule and the actual activity of the current anti-TB drugs as a consequence of their differences in their chemical structures. Second, the annotations of the molecules as active or inactive employed a classification approach instead of a regression one. Notice that, in contrast to their regression counterparts, classification approaches do not need to predict the exact values of a biological property in a dataset, and, therefore, they do not need to deal so much with the potentially great uncertainty of the data. Third, the chosen MIC_90_ cutoffs prevented (as much as it was possible) the imbalance between the number of molecules labeled as active and the number of molecules considered inactive. Fourth, the MIC_90_ cutoffs are in the range (some of them are lower and, therefore, more rigorous) of the cutoffs selected in high-throughput screening campaigns to prioritize chemicals with potent anti-TB activity [49]. Fifth, from a phenomenological point of view, even though several anti-TB drugs appear in our dataset, the purpose of our mtc-QSAR-EL was to perform virtual screening of large and heterogenous external datasets to identify novel molecular patterns different from those present in the current anti-TB drugs.

The SMILES codes of the 1571 cases were stored in a file of the type *.smi. Following this, we used the software OpenBabel v2.4.0 [50] to convert this file to *.sdf, obtaining the connectivity table for each chemical present in our dataset; no additional standardization actions were applied. Then, using the sdf file as input, we employed the software QuBiLS-MAS v1.0 to compute the molecular descriptors known as local atom-based stochastic quadratic indices *LQI* [51,52]. These are topological descriptors with successful applications in medicinal chemistry and drug discovery [53,54]. We calculated the *LQI* descriptors of order k (with k from 0 to 5) by using predefined parameters such as algebraic form (quadratic), constraints (atom-based), matrix type (stochastic), cutoff (keep all), groups (local—referring to specific atom types such as aliphatic and aromatic carbons, methyl groups, halogens, and heteroatoms), and aggregation operator (Manhattan distance). These *LQI* descriptors were weighted by atomic properties such as hydrophobicity (*HYD*), atomic weight (*AW*), polarizability (*POL*), polar surface area (*PSA*), and Kupchick vertex degree (*KU*).

Notice that the *LQI* descriptors are not able to discriminate the effect of the chemical structure of a molecule on the anti-TB activity when the experimental condition *cj* changes, e.g., use of different assay times (*ta*), *Mtb* strains (*bs*), and/or test protocols (*ap*). This means that the *LQI* descriptor for a molecule will have the same value regardless of the experimental condition *cj* used to assess the anti-TB activity of that molecule. To solve this inconvenience, we applied the adaptation of the Box–Jenkins approach, which is a distinctive characteristic of all the PTML models [18,19,20,21,22,23,24,25,26,27,28,29,30,31,32,33,34,55]:

(1) $$avg\left[LQI\right]c_{j}=\frac{1}{n\left(cj\right)}\times \sum_{i=1}^{n\left(cj\right)}LQI_{i}\;\;$$

In Equation (1), the term *avg*[*LQI*]*cj* is the average of the *LQI_i_* values for all the molecules in the training set, which were annotated as active by considering the same experimental condition *cj*. Consequently, *n*(*cj*) refers to the number of molecules labeled as active (also in the training set) that were tested under the same *cj*. Notice that, as mentioned before, the experimental condition *cj* depends on the elements *ta*, *bs*, and *ap*, and, therefore, Equation (1) was applied to each of the elements of *cj* separately. For instance, if *cj* = *ta*, then *avg*[*LQI*]*cj* = *avg*[*LQI*]*ta* and *n*(*cj*) = *n*(*ta*), meaning that, in this case, Equation (1) was employed for calculations depending only on the assay times. The same line of thinking was applied to the elements *bs* and *ap*. In the second step of the Box–Jenkins approach,

(2) $$D\left[LQI\right]cj=\left[\frac{LQI-avgLQI\left(cj\right)}{SDev\left(LQI\right)}\right]\cdot \sqrt{p\left(cj\right)}\;\;\;$$

In Equation (2), *D*[*LQI*]*cj* is a multi-target descriptor that considers both the chemical structure of a molecule and a specific element of the experimental condition *cj*. Therefore, as in the case of Equation (1), Equation (2) was applied to each element of *cj* separately. The descriptor *D*[*LQI*]*cj* characterizes how much any molecule physicochemically and structurally deviates from a group of molecules annotated as active, having been tested by considering the same element of *cj*. Additionally, *SDev*[*LQI*] is the standard deviation calculated from all the values of each *LQI* descriptor for the molecules/cases present only in the training set. Last, the term *p*(*cj*) is the a priori probability calculated as the quotient of the number of molecules/cases in the training set assayed by involving a given element of *cj* and the total number of compounds present in the training set.

### 3.2. Building the Mtc-QSAR-EL Model

When generating the mtc-QSAR-EL model, we followed a series of steps. First, we divided our dataset (containing 1571 cases) into training and test sets. In this sense, for each assay time, we sorted the molecules in ascending order of their MIC_90_ values. After, within each assay time, the first three molecules/cases were annotated as members of the training set, while the fourth molecule/case was labeled to belong to the test set. Such a ratio of 3:1 was repeated in the whole dataset. Thus, the training set was formed by 1184 molecule/cases (75.37% of the dataset), 602 active and 582 inactive; the training set was employed to find the best mtc-QSAR-EL model. The test set (the remaining 24.63%) contained 387 molecules/cases, 201 considered active and 186 defined as inactive; the test set was used to estimate the predictive power of the mtc-QSAR-EL model.

We employed the software IMMAN v1.0 [56], which allowed us to prioritize those *D*[*LQI*]*cj* descriptors that were likely to exhibit the highest discriminatory power. In doing so, we used two information theory metrics: the differential Shannon entropy [57] and the information gain ratio [58]. We ranked the *D*[*LQI*]*cj* descriptors according to their decreasing values of the geometric mean between the two aforementioned metrics. While selecting the *D*[*LQI*]*cj* descriptors with the highest discriminatory power (high geometric mean values), we estimated the redundancy among them by computing the pairwise Pearson correlation coefficient (*PCC*) [59]. Only the *D*[*LQI*]*cj* descriptors with pairwise correlation values in the interval −0.7 < *PCC* < 0.7 were chosen to construct the mtc-QSAR-EL model.

We should recall that the mtc-QSAR-EL model is an ensemble of artificial neural networks (ANNs), and, thus, to form such an ensemble, we searched for the best ANNs, all of them being based on the multi-layer perceptron (MLP) architecture. When selecting the most appropriate MLP networks, we examined, in a first step, global statistical metrics such as accuracy [*Ac*(%)], Matthews’ correlation coefficient (*MCC*) [60], sensitivity [*Sn*(%)], and specificity [*Sp*(%)]. However, ultimately, the best mtc-QSAR-EL model was chosen as an ensemble of those MLP networks that exhibited the highest values of the sensitivities [*Sn*(%)]*ta*, [*Sn*(%)]*bs*, and [*Sn*(%)]*ap*, as well as the specificities [*Sp*(%)]*ta*, [*Sp*(%)]*bs*, and [*Sp*(%)]*ap*. Notice that these six local statistical indices depended on specific elements of the experimental condition *cj*. The ANN package of the software STATISTICA v13.5.0.17 [61] was employed to generate the MLP networks and subsequently build the mtc-QSAR-EL model.

### 3.3. Applicability Domain

When defining the applicability domain (AD) of the mtc-QSAR-EL model, we used two approaches. One of them was the consensus predictions since our mtc-QSAR-EL model was an ensemble of different MLP networks [62,63]. This means that for each molecule present in our dataset, the predicted probabilities given by each of the MLP networks were averaged, resulting in the probability of the ensemble (mtc-QSAR-EL) model for that molecule. As the second method to estimate the AD, we used a modification of the descriptors’ space approach reported by Speck-Planche [64]. In doing so, for each molecule present in the dataset containing the 1571 cases/molecules, we generated the local scores (LS) of the applicability domain for each *D*[*LQI*]*cj* descriptor in the following manner. If for a specific *D*[*LQI*]*cj* descriptor, the *D*[*LQI*]*cj* value of a query molecule fell between the maximum and minimum *D*[*LQI*]*cj* values, the LS took the value of one; otherwise, the LS was equal to zero. This operation was repeated for each *D*[*LQI*]*cj* descriptor that entered in the mtc-QSAR-EL model. Then, the metric known as the total score of applicability domain (TSAD) was calculated for the aforementioned query molecule as the sum of the LS values. In the end, in magnitude, the maximum value of TSAD was equal to the number of *D*[*LQI*]*cj* descriptors present in the mtc-QSAR-EL model.

### 3.4. Interpretation of the Molecular Descriptors in the Mtc-QSAR-EL Model

Due to the non-linear nature of the mtc-QSAR-EL model, we provided the physicochemical and structural interpretation of the *D*[*LQI*]*cj* descriptors by strictly following the guidelines recently reported by Speck-Planche and co-workers [21,64]. In this sense, we used the sensitivity values *SV* calculated by the ANN package of the software STATISTICA v13.5.0.17 to rank the different *D*[*LQI*]*cj* descriptors in terms of their importance in the mtc-QSAR-EL model while calculating the class-based means to help us gain insights regarding how the values of the *D*[*LQI*]*cj* descriptors should vary (increase or decrease) to enhance both the anti-TB activity and the versatility of a molecule to inhibit more than one *Mtb* strain.

### 3.5. Virtual Screening of Agency-Regulated Chemicals

We virtually screened a large and heterogeneous dataset formed by 8898 agency-regulated chemicals. None of these molecules were present in the dataset used to build the mtc-QSAR-EL model (1571 chemicals/cases). We predicted each of the 8898 agency-regulated chemicals under the 24 experimental conditions *cj* reported in Table 6. Then, we generated the metric FA(%), which expressed the percentage of experimental conditions *cj* in which each of these 8898 agency-regulated chemicals was predicted as active (anti-TB agent) by the mtc-QSAR-EL model (Supplementary Material 8). We also defined a second metric symbolized as S(TSAD); this was calculated as the sum of all the TSAD values reported for a single agency-regulated chemical by considering the 24 experimental conditions *cj*. The meaning of S(TSAD) was that the higher its value, the more reliable the predictions associated with that agency-regulated chemical in terms of whether it fell within the AD of the mtc-QSAR-EL model by considering the 24 experimental conditions *cj* (Supplementary Material 8). By using FA(%) and S(TSAD), we could rank the agency-regulated chemicals according to their predicted anti-TB activity and the reliability of the predictions, respectively.

## 4. Conclusions

The search for novel and more efficient anti-TB therapies can be greatly accelerated by employing in silico models as tools in the context of computational drug repurposing, where the prioritized hits could then be experimentally validated. Current computer-aided drug discovery approaches should, however, focus on including more information regarding the experimental conditions under which the molecules are assayed. By doing so, computational models will be able to make more accurate predictions while also providing a deeper phenomenological understanding of the physicochemical properties and structural requirements associated with the potentiation of the anti-TB activity and versatility to inhibit different *Mtb* strains. The mtc-QSAR-EL model created in this work constitutes an advance in early drug discovery against TB, demonstrating that it is possible to identify anti-TB agents and/or multi-strain inhibitors of *Mtb* via virtual screening while proposing new molecular patterns that could be promising as starting points for anti-TB drug development. The present report confirms the promising applications of the PTML modeling methodology, which can be extended to diverse research areas devoted to finding treatments for unmet needs.

## Figures and Tables

**Table 1:** Descriptors of the type *D*[*LQI*]*cj* which entered in the mtc-QSAR-EL model.

Symbol	Definition
*D*[*LASSq*3(*HYD*)*G*]*ta*	Deviation of the atom-based stochastic quadratic index of order 3, weighted by the hydrophobicity of the halogens and their chemical environments, and depending on the chemical structure and the assay time.
*D*[*LASSq*0(*POL*)*G*]*ta*	Deviation of the atom-based stochastic quadratic index of order 0, weighted by the polarizability of the halogens, and depending on the chemical structure and the assay time.
*D*[*LASSq*0(*HYD*)*Y*]*ta*	Deviation of the atom-based stochastic quadratic index of order 0, weighted by the hydrophobicity of the heteroatoms, and depending on the chemical structure and the assay time.
*D*[*LASSq*1(*PSA*)*Y*]*ta*	Deviation of the atom-based stochastic quadratic index of order 1, weighted by the polar surface area of the heteroatoms and their adjacent atoms, and depending on the chemical structure and the assay time.
*D*[*LASSq*0(*HYD*)*M*]*bs*	Deviation of the atom-based stochastic quadratic index of order 0, weighted by the hydrophobicity of the methyl groups, and depending on the chemical structure and the *Mtb* strain against which the molecule was tested.
*D*[*LASSq*0(*AW*)*P*]*bs*	Deviation of the atom-based stochastic quadratic index of order 0, weighted by the atomic weight of the aromatic carbons, and depending on the chemical structure and the *Mtb* strain against which the molecule was tested.
*D*[*LASSq*2(*HYD*)*Y*]*bs*	Deviation of the atom-based stochastic quadratic index of order 2, weighted by the hydrophobicity of the heteroatoms and their chemical environments, and depending on the chemical structure and the *Mtb* strain against which the molecule was tested.
*D*[*LASSq*2(*HYD*)*G*]*ap*	Deviation of the atom-based stochastic quadratic index of order 2, weighted by the hydrophobicity of the halogens and their chemical environments, and depending on the chemical structure and information regarding the assay protocol.
*D*[*LASSq*5(*AW*)*G*]*ap*	Deviation of the atom-based stochastic quadratic index of order 5, weighted by the atomic weight of the halogens and their chemical environments, and depending on the chemical structure and information regarding the assay protocol.
*D*[*LASSq*3(*KU*)*G*]*ap*	Deviation of the atom-based stochastic quadratic index of order 3, weighted by Kupchick’s vertex degrees of the halogens and their chemical environments, and depending on the chemical structure and information regarding the assay protocol.
*D*[*LASSq*2(*HYD*)*M*]*ap*	Deviation of the atom-based stochastic quadratic index of order 2, weighted by the hydrophobicity of the methyl groups and their chemical environments, and depending on the chemical structure and information regarding the assay protocol.
*D*[*LASSq*2(*AW*)*P*]*ap*	Deviation of the atom-based stochastic quadratic index of order 2, weighted by the atomic weight of the aromatic carbons and their chemical environments, and depending on the chemical structure and information regarding the assay protocol.

**Table 2:** Parameters characterizing the different MLP networks that were employed to build the mtc-QSAR-EL model.

Network Notation ^a^	Training Algorithm ^b^	Error Function ^c^	Hidden Activation ^d^	Output Activation
MLP 12-45-2	BFGS 116	Entropy	Tanh	Softmax
MLP 12-33-2	BFGS 138	Entropy	Logistic	Softmax
MLP 12-41-2	BFGS 104	SOS	Tanh	Logistic

^a^ The number of input nodes appears before the first hyphen, the number of neurons in the hidden layer appears between the two hyphens, and the number appearing after the second hyphen indicates the number of classes that were predicted by the mtc-QSAR-EL model. ^b^ BFGS—Broyden–Fletcher–Goldfarb–Shanno algorithm; the number accompanying the symbol BFGS is the number of epochs used to train the MLP networks. ^c^ SOS—sum of squares. ^d^ Tanh—hyperbolic tangent.

**Table 3:** Statistical metrics indicating the global performance of the mtc-QSAR-EL model.

SYMBOLS ^a^	Training Set	Test Set
***N*_Active_**	602	201
***CC*_Active_**	572	172
***Sn*(%)**	95.02%	85.57%
***N*_Inactive_**	582	186
***CC*_Inactive_**	534	160
***Sp*(%)**	91.75%	86.02%
***MCC***	0.869	0.716

^a^ *N*_Active_—number of molecules labeled as active; *N*_Inactive_—number of molecules annotated as inactive; *CC*_Active_—number of molecules rightly classified/predicted as active; *CC*_Inactive_—number of molecules correctly classified/predicted as inactive; *Sn*(%)—sensitivity (percentage of molecules properly classified as active); *Sp*(%)—specificity (percentage of molecules properly classified as inactive); *MCC*—Matthews’ correlation coefficient.

**Table 4:** Tendencies of variation calculated for the different molecular descriptors in the mtc-QSAR-EL model according to the class-based mean approach.

Symbol	Class-Based Means		Tendency ^a^
	Active	Inactive	
*D*[*LASSq*3(*HYD*)*G*]*ta*	−1.1976 × 10^−4^	−2.0514 × 10^−2^	Increase
*D*[*LASSq*0(*POL*)*G*]*ta*	5.0005 × 10^−3^	6.3674 × 10^−3^	Decrease
*D*[*LASSq*0(*HYD*)*Y*]*ta*	5.1000 × 10^−3^	−4.4105 × 10^−1^	Increase
*D*[*LASSq*1(*PSA*)*Y*]*ta*	1.2460 × 10^−3^	−1.6800 × 10^−1^	Increase
*D*[*LASSq*0(*HYD*)*M*]*bs*	1.4621 × 10^−2^	−3.0065 × 10^−1^	Increase
*D*[*LASSq*0(*AW*)*P*]*bs*	8.1935 × 10^−3^	−2.2074 × 10^−1^	Increase
*D*[*LASSq*2(*HYD*)*Y*]*bs*	−4.0897 × 10^−3^	−2.1845 × 10^−1^	Increase
*D*[*LASSq*2(*HYD*)*G*]*ap*	1.0793 × 10^−2^	−2.4162 × 10^−1^	Increase
*D*[*LASSq*5(*AW*)*G*]*ap*	1.2911 × 10^−2^	−1.8708 × 10^−1^	Increase
*D*[*LASSq*3(*KU*)*G*]*ap*	9.6900 × 10^−3^	−1.1239 × 10^−1^	Increase
*D*[*LASSq*2(*HYD*)*M*]*ap*	1.6609 × 10^−2^	−2.8205 × 10^−1^	Increase
*D*[*LASSq*2(*AW*)*P*]*ap*	1.1549 × 10^−2^	−2.8297 × 10^−1^	Increase

^a^ This denotes how the value of a *D*[*LQI*]*cj* descriptor should vary (increase or diminution) in order for a molecule to enhance its anti-TB activity and versatility (ability to inhibit more than one *Mtb* strain).

**Table 5:** Top-ranked regulated chemicals identified by mtc-QSAR-EL as potential multi-strain inhibitors against *Mtb* (MIC_90_ < 7622.22 nM) and predicted by the webserver mycoCSM.

ID ^a^	Name	FA(%) ^b^	S(TSAD) ^c^	log10(MIC) ^d^	MIC (nM) ^e^
CHEMBL78535	Ancriviroc	100.00	288	−4.624	23768.40
CHEMBL1450565	Aklomide	100.00	279	−4.128	74473.20
CHEMBL2104712	Nisterime acetate	100.00	279	−4.673	21232.44
CHEMBL2106822	Nizofenone	95.83	288	−4.332	46558.61
CHEMBL2104159	Bromoxanide	100.00	276	−4.838	14521.12
CHEMBL1199080	Bretylium	95.83	279	−3.91	123026.88
**CHEMBL3330226**	**Macozinone**	**95.83**	**279**	**−7.558**	**27.67**
CHEMBL1909324	Pinaverium	95.83	279	−4.668	21478.30
CHEMBL292702	Maitansine	95.83	279	−4.776	16749.43
CHEMBL2111120	Nitralamine	95.83	276	−4.02	95499.26
CHEMBL289832	Licostinel	95.83	276	−3.826	149279.44
**CHEMBL1448**	**Niclosamide**	**95.83**	**276**	**−5.249**	**5636.38**
CHEMBL2104616	Clonitazene	91.67	288	−4.866	13614.45
**CHEMBL1269025**	**Edoxaban**	**91.67**	**288**	**−5.731**	**1857.80**
CHEMBL2106056	Cronidipine	91.67	288	−4.771	16943.38
**CHEMBL1241348**	**Faldaprevir**	**91.67**	**288**	**−5.275**	**5308.84**
**CHEMBL2013174**	**Vedroprevir**	**91.67**	**288**	**−5.446**	**3580.96**
CHEMBL2104730	Nitroxinil	91.67	279	−3.881	131522.48
CHEMBL493636	Sulfanitran	91.67	279	−4.678	20989.40
CHEMBL9484	Clofilium	91.67	279	−4.042	90782.05
**CHEMBL1822872**	**BTZ-043**	**91.67**	**279**	**−7.01**	**97.72**
CHEMBL1178725	Nolpitantium	91.67	279	−4.876	13304.54
**CHEMBL2104390**	**Ilatreotide**	**91.67**	**279**	**−5.123**	**7533.56**
CHEMBL56367	Nimesulide	87.50	288	−4.907	12387.97
CHEMBL2107448	Loprazolam	87.50	288	−4.945	11350.11
**CHEMBL3670800**	**ALK-4290**	**87.50**	**288**	**−5.298**	**5035.01**
**CHEMBL1181731**	**Teglarinad**	**87.50**	**288**	**−5.154**	**7014.55**
CHEMBL1170047	Iniparib	87.50	279	−4.117	76383.58
CHEMBL491	Hydroxyflutamide	87.50	279	−4.221	60117.37
CHEMBL452	Clonazepam	87.50	279	−4.223	59841.16
CHEMBL1274	Nilutamide	87.50	279	−4.559	27605.78
CHEMBL2110930	Fubrogonium	87.50	279	−4.125	74989.42
CHEMBL2110825	Dodeclonium	87.50	279	−4.19	64565.42
CHEMBL397647	JNJ-17166864	87.50	279	−4.53	29512.09
CHEMBL2105721	Nivocasan	87.50	279	−4.644	22698.65
CHEMBL2107326	Dasantafil	87.50	279	−5.015	9660.51
CHEMBL1908326	Meclonazepam	83.33	288	−4.281	52360.04
CHEMBL1823817	CE-224535	83.33	288	−4.736	18365.38
**CHEMBL1276663**	**Cefozopran**	**83.33**	**288**	**−5.143**	**7194.49**
CHEMBL2110800	Ciclonium	83.33	279	−4.48	33113.11
CHEMBL1337	Nitisinone	83.33	279	−4.569	26977.39
CHEMBL512306	[18F]D	83.33	279	−4.532	29376.50
CHEMBL435191	Edotecarin	83.33	279	−4.521	30130.06
CHEMBL2074922	Efonidipine	83.33	279	−4.894	12764.39

^a^ Highlighted chemicals are those predicted by the mtc-QSAR-EL model and the webserver mycoCSM to have MIC_90_ < 7622.22 nM. ^b^ FA(%)—percentage of times that a chemical was predicted as active (anti-TB agent) by the mtc-QSAR-EL model. ^c^ S(TSAD)—sum of all the TSAD values for a given chemical by considering all the experimental conditions reported in this study. ^d^ log10(MIC)—predicted value of anti-TB activity estimated by the webserver mycoCSM. ^e^ Minimum inhibitory concentration calculated from the predicted log10(MIC).

**Table 6:** Experimental conditions *cj* (combinations of the elements *ta*, *bs*, and *ap*) reported in the present work.

MIC_90_ Cutoff Value (nM) ^a^	*ta* ^b^	*bs* ^c,d^	*ap* ^e^
<1146.75	10d	*Mtb* (H37Rv_NRF)	LORA method
	10d	*Mtb* (H37Rv)	Spectrophotometric assay (OD580-OD600)
≤1500	14d	*Mtb* (H37Rv)	Broth dilution method
<7622.22	3d	*Mtb* (H37Rv_NRF)	Spectrophotometric assay (OD580-OD600)
	3d	*Mtb* (MC2 6220_NRF)	Spectrophotometric assay (OD580-OD600)
	3d	*Mtb* (MC2 6220_RF)	Spectrophotometric assay (OD580-OD600)
<5829.77	4d	*Mtb* (H37Rv)	AlamarBlue/Resazurin/MABA method
≤5300	5d	*Mtb* (H37Rv)	AlamarBlue/Resazurin/MABA method
	5d	*Mtb* (H37Rv_ATCC 25618)	Spectrophotometric assay (OD580-OD600)
	5d	*Mtb* (H37Rv)	Spectrophotometric assay (OD580-OD600)
	5d	*Mtb* (INH-R)	AlamarBlue/Resazurin/MABA method
	5d	*Mtb* (H37Rv_ATCC 27294)	Broth dilution method
≤5000	6d	*Mtb* (H37Rv_ATCC 25618)	AlamarBlue/Resazurin/MABA method
	6d	*Mtb* (H37Rv)	AlamarBlue/Resazurin/MABA method
	6d	*Mtb* (MC2 6220_NRF)	Spectrophotometric assay (OD580-OD600)
≤4940	7d	*Mtb* (H37Rv)	AlamarBlue/Resazurin/MABA method
	7d	*Mtb* (H37Rv_ATCC 27294)	AlamarBlue/Resazurin/MABA method
	7d	*Mtb* (INH-R)	AlamarBlue/Resazurin/MABA method
	7d	*Mtb* (H37Rv_ATCC 27294)	Broth dilution method
	7d	*Mtb* (H37Rv)	Broth dilution method
	7d	*Mtb* (RMP-R)	AlamarBlue/Resazurin/MABA method
	7d	*Mtb* (H37Rv)	Spectrophotometric assay (OD580-OD600)
	7d	*Mtb* (MC2 6220_RF)	Spectrophotometric assay (OD580-OD600)
	7d	*Mtb* (MC2 6220_NRF)	Spectrophotometric assay (OD580-OD600)

^a^ Activity value from which a molecule was considered active. ^b^ *ta*—assay time. ^c^ *bs*—bacterial strain belonging to *Mtb*. ^d^ The following abbreviations were used: RF (non-replicative form), RF (replicative form), INH-R (isoniazid-resistant strain), and RMP-R (rifampicin-resistant strain). ^e^ *ap*—information regarding the assay protocol.

## Data Availability

All the chemical and biological (raw) data were retrieved from the public repository known as ChEMBL (https://www.ebi.ac.uk/chembl/, accessed on 23 June 2021).

## Supplementary Materials

The following are available online at https://www.mdpi.com/article/10.3390/antibiotics10081005/s1. Supplementary Material 1: Chemical and biological data including the *LQI* descriptors and the averages. Supplementary Material 2: *D*[*LQI*]*cj* descriptors, classification results, and local metrics. Supplementary Material 3: Applicability domain of the mtc-QSAR-EL model. Supplementary Material 4: Chemical data of dataset containing the 8898 agency-regulated chemicals and their *LQI* descriptors. Supplementary Material 5: *D*[*LQI*]*cj* descriptors calculated for the 8898 agency-regulated chemicals. Supplementary Material 6: Prediction results for the 8898 agency-regulated chemicals under multiple experimental conditions. Supplementary Material 7: Applicability domain of the predictions involving the 8898 agency-regulated chemicals. Supplementary Material 8: Calculation of the metrics FA(%) and S(TSAD).
